# Hepatic endoplasmic reticulum calcium fluxes: effect of free fatty acids and K_ATP_ channel involvement

**DOI:** 10.1042/BSR20202940

**Published:** 2021-02-01

**Authors:** Rawan Al-Rawi, Xudong Wang, Kenneth McCormick

**Affiliations:** Department of Pediatrics, University of Alabama at Birmingham, Birmingham, AL, U.S.A.

**Keywords:** ER stress, sarco/endoplasmic reticulum Ca+2 – ATPase (SERCA), polyunsaturated fatty acids (PUFA)

## Abstract

As a common sequel to obesity, plasma and intracellular free fatty acid (FFA) concentrations are elevated and, as a consequence, manifold disturbances in metabolism may ensue. Biochemical processes in the cytosol and organelles, such as mitochondria and endoplasmic reticulum (ER), can be disturbed. In the ER, the maintenance of a high calcium gradient is indispensable for viability. In sarcoplasmic reticulum, selective FFA can induce ER stress by disrupting luminal calcium homeostasis; however, there are limited studies in hepatic microsomes. Our studies found that FFA has a noxious effect on rat hepatic microsomal calcium flux, and the extent of which depended on the number of double bonds and charge. Furthermore, insofar as the FFA had no effect on microsomal calcium efflux, their inhibitory action primarily involves calcium influx. Finally, other cationic channels have been found in hepatic ER, and evidence is presented of their interaction with the Ca^2+^ ATPase pump.

## Introduction

One function of the endoplasmic reticulum (ER) is to properly assemble proteins that are eventually destined for intracellular organelles, cytoplasm, or the cell surface [1]. In liver, the ER is the primary calcium reservoir [2] and, as such, governs the activity of multiple enzymes and chaperones involved in protein folding and ER function. To accomplish these tasks, high Ca^2+^ levels in the ER are a requisite [3]. Hence, in the ER, there is a thousand-fold calcium gradient over the cytosol [2,4]. The microsomal concentration of Ca^2+^ is regulated by the coordination of contradictory fluxes across the membrane: Ca^2+^ influx from the cytosol to the ER occurs mainly via the P2-ATPase ion transporter family, whereas efflux transpires through various tightly regulated ion channels [2,5].

Sarcoplasmic reticulum Ca^2+^-ATPase (SERCA) is a Ca^2+^-transporter ATPase, the function of which is the uptake of Ca^2+^ from cytosol into the ER lumen. The chief isoform in the liver is SERCA2b [6,7]. Studies have shown that SERCA2b protein and mRNA levels were significantly decreased (30%) in the liver of obese mice. Moreover, SERCA2b expression was 50% reduced in high-fat diet fed mice vs normal fat diet fed mice. In contrast, SERCA2b overexpression reduces liver triglyceride level by 25%, reduces lipogenic gene expression levels, and free fatty acid (FFA) synthase by 73% [3].

Chronic ER stress has been coupled to the etiology of numerous obesity-related diseases such as glucose intolerance and insulin resistance, with the longing that ER calcium fluxes, either entry or exit, may eventually be therapeutic targets [3].

In diverse cell types, chronic exposure to increased levels of FFA leads to ER stress with the prospect of resultant cell death. The underlying mechanism that promotes ER stress in response to FFA in hepatocytes is not fully understood. But based on several observations in other cell types, it is plausible that depletion of ER calcium by FFA may beget hepatic ER stress [1].

In eukaryotic cells, membrane lipids can exist in various liquid gel-like states. Altered lipid composition of cell membranes may impair protein function leading to numerous pathologies such as obesity, DM, and hyperlipidemia [8,9]. One physicochemical property of cellular membranes, colloquially termed fluidity, denotes the non-static fluxive state of the lipid-rich membranes. This function depends primarily on the lipid composition and phospholipid interactions and can also be modified by temperature and osmolarity [10]. Plasma membrane fluidity of human neuroblastoma cells is especially enhanced by unsaturated fatty acids with four or more double bonds [11]. Of note, the ER membrane, compared with the plasma membrane, is more fluid given the relative paucity of saturated fatty acids and cholesterol. At room temperature, cholesterol and saturated FFA impede fluidity by increasing membrane cohesion and packing of adjacent lipids, rendering the membrane more rigid [12,13], decreased fluidity can inactivate Ca^2+^ ATPase and promote unfolded protein stress [14]. In an epidermoid carcinoma cell line (A 431), reducing membrane cholesterol content enhances epidermal growth factor (EGF) receptor phosphorylation [8]. Moreover, maintenance of cell membrane fluidity is crucial for optimal function of membrane-embedded proteins. In contrast, by reducing intermolecular Van der Waals forces between phospholipid tails, unsaturated fatty acids render the membrane less rigid.

In rat hepatic microsomes, we explored the effect of FFA of various carbon chain lengths and degrees of saturation on the regulation of net ER calcium uptake. Moreover, the effect of altering ER membrane fluidity, done so by manipulating the osmolality or cholesterol content, was also investigated.

Finally, based on manipulating the activity of a purported hepatic K_ATP_ channel, this conduit interacts with the Ca^2+^ ATPase pump, with or without the presence of FFA.

## Materials and methods

^45^CaCl_2_ was purchased from American Radiolabeled Chemicals. Other compounds and general chemicals were from Sigma–Aldrich.

### Preparation of rat liver microsomes

Microsomes were isolated from the liver of Sprague–Dawley male rats (150–200 g body weight) as previously described [15,16]. All animal procedures were undertaken with approval and oversight of the Institutional Animal Care and Use Committee (IACUC) at the University of Alabama Birmingham. Animals were killed by carbon dioxide. Tissue samples were homogenized in an ice‐bath with 4 vol. of 0.25 mol/l sucrose and 50 mmol/l Tris/HCl, pH 7.3. The homogenate was centrifuged for 10 min at 1000×***g***. The supernatant portion was removed and centrifuged for 20 min at 10000×***g***. and, thereafter, centrifuged for 60 min at 100000×***g***. The resulting pellet was washed twice with the same buffer. The microsomes were resuspended at a protein concentration of 15–20 mg/ml (measured using a Bio‐Rad protein assay). All animal work took place at University of Alabama labs.

### Calcium release

Rat liver microsomes were washed with 6% PEG in water, centrifuged at 1800×***g*** for 30 s, and resuspended with 15 mg/ml in MOPS-KCl buffer containing 20 mM MOPS, 100 mM KCl, 20 mM NaCl, 1 mM MgCl_2_, pH 7.2. Then, to it 1 mM CaCl_2_ and 40 μCi/ml ^45^CaCl_2_ were added, incubated for 1 h at room temperature. Ca^2+^ efflux was initiated by a 100-fold dilution of the microsomes into MOPS-KCl buffer (with or without FA); 0.2 ml of microsomes were placed on 0.45-μm filter under vacuum at various times, and rinsed with a total of 2 ml ice-cold 0.25 M sucrose buffer with 60 mM Tris/HCl (pH 7.2) containing 1 mM LaCl_3_. Total counts per 0.2 ml diluted microsome suspension: 100552 cpm [17].

### Calcium ATPase activity

Ca^2+^-ATPase activity of liver microsomes was measured with a direct colorimetric assay. The reaction was initiated by adding 0.1 mg/ml liver microsomes to the assay buffer containing 0.2 mM EGTA, 1 mM ATP, 5 mM NaN_3_, 2.5 μM Ruthenium Red, and 0.25 mM CaCl_2_ to give 20 μM free calcium in buffer of 100 mM KCl and 50 mM imidazole-HCl, pH 6.8. After 15-min incubation at 37°C, 50 μl of reaction was added to 200 μl dye reagent (Malachite Green), the absorbance of the color complex was measured at 630 nm with a microplate reader (Bio-Rad). Pi quantity was determined from a standard curve prepared with known amounts of KH_2_PO_4_; the reaction was linear to at least 12.5 nmols Pi (Kwok-Ming Chan, Analytical Biochemistry 157:375-380, 1986).

### Net calcium uptake

**Figure 1:**
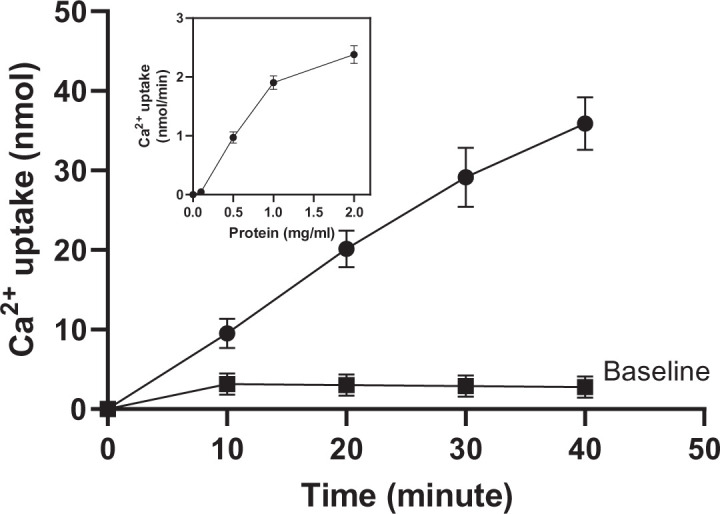
Calcium uptake in rat liver microsomes The assay was conducted using 100 μM free calcium and 0.1 μCi/ml of ^45^CaCl_2_ in a total volume of 0.4 ml at 37°C and initiated with the addition of the microsomes (0.1 mg of protein/ml). Uptake was linear for more than 30 min. Furthermore, the uptake was dependent on the presence of ATP in the assay, as evidenced by the baseline data (no ATP). Data are presented as mean ± SEM (*n*=3). Insert graph demonstrates the uptake rate dependence on protein concentration

Calcium uptake was measured in the following medium: 100 mmol/l KCl, 10 mmol/l MOPS buffer, pH 7.2, 5 mmol/l sodium azide, 5 mmol/l MgCl_2_, 1 mmol/l ATP, 5 mmol/l creatine phosphate, 5 units/ml creatine phosphokinase, 5 mmol/l ammonium oxalate, 100 μM free calcium, and 0.1 μCi/ml of ^45^CaCl_2_ in a total volume of 0.4 ml. The assay was conducted at 37°C and initiated with the addition of the microsomes to a concentration of 0.1 mg of protein/ml. The reactions were terminated after 30 min by filtering through 0.45-μm membrane filters (Millipore Corp.) and the filters were washed with 0.25 mol/l sucrose (2 ml). The filters were dried and ^45^Ca^2+^ was determined using liquid scintillation counter (PerkinElmer) [16]. Figure 1 displays the linearity of Ca^+2^ uptake (for more than 30 min) and the assay dependence on ATP.

### Microsomal membrane cholesterol alteration

To reduce ER membrane cholesterol and reduce membrane fluidity, rat liver microsomes (2 mg/ml) were treated with 25 mM methyl-b-cyclodextrin (MBC) for 30 min at 37°C, centrifuged at 20000×**g** for 5 min, and resuspended in buffer.

In contrast, to increase membrane rigidity, that is, reduce fluidity, a water-soluble complex (1:1) of 1.5 mM cholesterol/MBC was added to the microsomes and incubated for another 30 min at 37°C [18].

### Statistical analysis

Data are presented as mean ± SEM (*n*). The significance of differences between groups was determined by Student’s *t* test and analysis of variance. A *P*<0.05 was deemed the value to reject the null hypothesis. All statistical analyses were performed with GraphPad Prism software (San Diego, CA, U.S.A.). Mean control values were nominally assigned a value of 100%.

## Results

### The effect of long-chain FFA carbon length and number of double bonds on net Ca^2+^ uptake in rat liver microsomes

**Figure 2:**
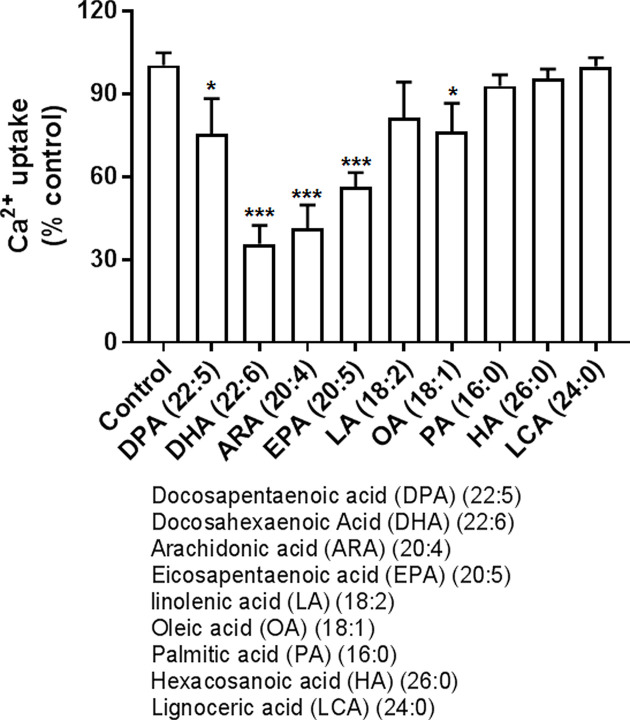
FFA effect on calcium uptake in rat liver microsomes Calcium uptake was measured at 37°C using 100 μM free calcium and 0.1 μCi/ml of ^45^CaCl_2_ in a total volume of 0.4 ml and initiated with the addition of the microsomes to a concentration of 0.1 mg of protein/ml. The effect of 50 μM long chain fatty acids with different carbon length and double bonds on Ca^2+^ uptake in rat liver microsomes. Calcium uptake was measured after 30-min incubation (see ‘Materials and methods’ section) in the presence/absence of FFA. The FFAs of different carbon length and double bonds were at 50 μM. Data presented as mean ± SEM (*n*=4). *P<0.05. ***P<0.0005 vs control.

The effect of FFA with different carbon lengths and double bond numbers was tested. Polyunsaturated FFA (PUFA) with four or more double bonds and more carbon atoms were most inhibitory [19]. However, even at 50 μM concentrations, the saturated fatty acids (>16 carbons) failed to significantly inhibit net calcium uptake (Figure 2). In general, the more double bonds present, the greater the inhibition. This finding is in concert with previous study in sarcoplasmic reticulum microsomes, namely, that unsaturated fatty acids are stronger inhibitors of ER calcium uptake than saturated fatty acids [20].

Binding of FFA to protein (e.g., albumin) is a concern by which the unbound calcium component can be altered. To wit, unsaturated FFA (oleic acid 18:1) can increase the calcium affinity constant for albumin binding compared with saturated FFA (stearic acid 18:0), however, the maximum Ca^+2^ binding only increases by 20%. Regardless, if protein–calcium binding were of concern, the saturated FFA would have caused, by reducing the free calcium concentration, more of a reduction in ER calcium uptake than the unsaturated FFA [21]. In any case, our assay did not contain albumin, only those proteins intrinsic to the ER.

### The effect of docosahexaenoic acid and arachidonic acid FFA on Ca^2+^ efflux and Ca^2+^ ATPase activity in rat liver microsomes

**Figure 3:**
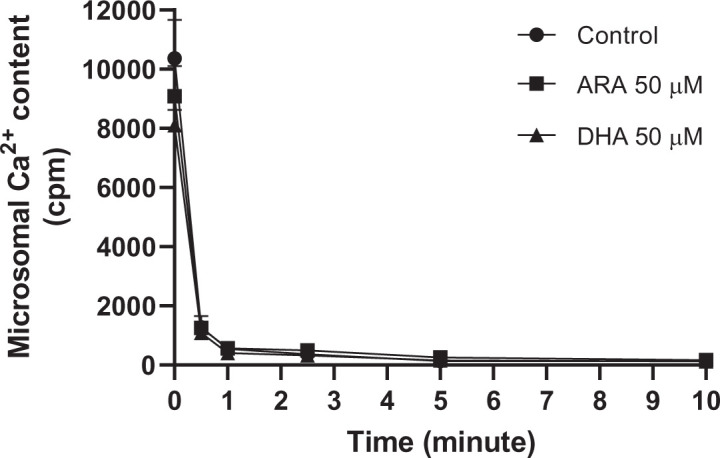
Effect of long-chain FFA on microsome Ca^2+^ content in rat liver microsomes The experiment was conducted using 1 mM CaCl_2_ and 40 μCi/ml ^45^CaCl_2_, incubated for 1 h at room temperature. The liver microsomes were incubated with 50 μM DHA or ARA, both of which inhibit net calcium uptake in microsomes (protein 15 mg/ml). Ca^2+^ efflux was initiated by a 100-fold dilution of the microsomes into MOPS-KCl buffer (with or without FA). Data are presented as mean ± SEM (*n*=3). There was no significant effect of FFA on calcium content over time (see ‘Materials and methods’ section). Recall that calcium uptake over a 1-min inhibition is negligible, as indicated in Figure 1.

The effect on Ca^2+^ efflux and Ca^2+^ ATPase of two common PUFAs, docosahexaenoic acid (DHA, 22:6, *n*=3) and arachidonic acid (ARA, 20:4, *n*=6) were investigated. Both fatty acids are abundant PUFAs in hepatic microsomal vesicles [22–24]. The effect of DHA or ARA at 50 μM (Figure 3) was explored at different time periods. Compared with control (no FFA added), Ca^2+^ efflux was not affected by either DHA or ARA [20]. Of note, the PUFA concentrations tested were lower than those applied in similar studies [1]. Also, a previous study found that Mg^+2^ decreased Ca^2+^ efflux caused by FFA from skeletal muscle endoplasmic vesicles. Thus, we tested efflux under varying Mg^+2^ concentrations (1–10 mM), yet no effect on Ca^2+^ efflux was discernible [20].

Given that the effect of FFA observed in these studies are acute, that is, over 5 min, infers that binding to the ER membrane on selective ion channels, rather than a change in membrane composition, is likely [25]. By determining net calcium leakage, the data incorporate all channel fluxes beside the well-known inositol triphosphate receptor (IP3) and ryanodine receptor (RyR) conduits. Such poorly described calcium routes include pannexins, presenilins, and those from the transient receptor potential family, if relevant, would have been in play in our assay [26].

**Figure 4:**
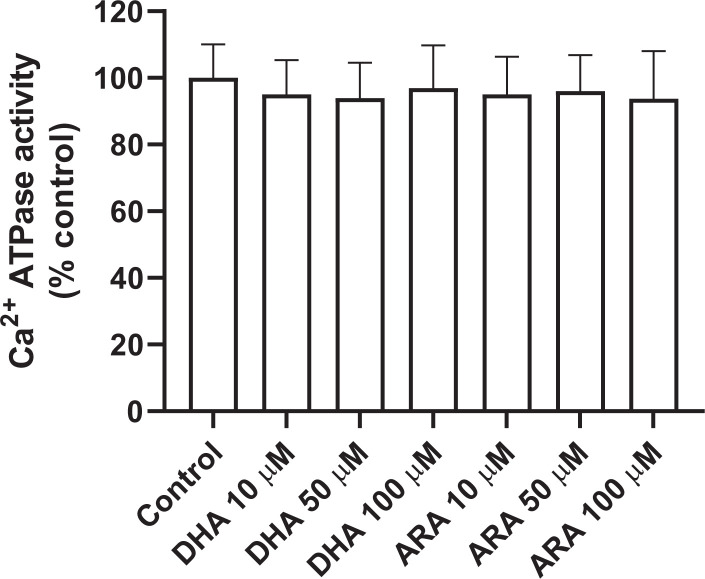
Effect of unsaturated FFA on Ca^2+^ -ATPase Different concentrations (10, 50, 100 μM) of two unsaturated fatty acids (DHA and ARA) were added to the Ca^2+^-ATPase assay containing 0.25 mM CaCl_2_ (20 μM free calcium) in buffer of 100 mM KCl and 50 mM imidazole-HCl. After 15-min incubation at 37°C, 50 μl of reaction was added to 200 μl dye reagent (malachite green), the absorbance of the color complex was measured at 630 nm with a microplate reader (Bio-Rad). Pi quantity was determined from a standard curve prepared with known amounts of KH_2_PO_4_; the reaction was linear to at least 12.5 nmols Pi. Control microsomes (protein 0.1 mg/ml) were not exposed to FFA, and designated as 100% in the figure. Control Ca^2+^-ATPase activity was 7.6 ± 1.1 nmol/min/mg. Data are presented as mean ± SEM (*n*=3). *P*>0.05 vs control for each FFA.

Lastly, hepatic ER Ca^2+^-ATPase activity upon exposure to different concentrations of DHA or ARA (10, 50, 100 μM) (Figure 4) was also examined—no sizable effect was observed, indicating that FFA inhibition of net Ca^2+^ uptake is not due to leakage of Ca^2+^ from the vesicles nor due to an attenuation in Ca^2+^-ATPase activity [20].

### K_ATP_ channel inhibition/activation effect on net ER calcium uptake and the FFA inhibitory effect

Both voltage-gated and inwardly rectifying K_ATP_ channels have been reported in hepatic ER [27–29]. And it has been opined that the potassium channel may alter ER calcium accumulation [27–29]. In particular, large-conductance K^+^ channels have been identified and, if dysfunctional, may promote ER stress [27]. Blocking the latter channel with 500 μM glibenclamide (GLY) alone led to a 22.7% reduction in calcium uptake, and this inhibition was 6.4% additive to that seen with 50 μM DHA. Antithetically, opening of the K_ATP_ channel with 500 μM diazoxide (DZX) alone produced a 12.3% increase in calcium uptake and blunted the FFA inhibition (Table 1).

**Table 1:** The effect of inhibitors of the K_ATP_ channels on net ER calcium uptake

Modifiers	Percent change versus control	Statistical significant versus control
DHA alone	–58.7%	*P*<0.01
DHA + GLY	–65.1%	*P*<0.01
DHA + GLY + DZX	–53.8%	*P*<0.01
DHA + DZX	–55.4%	*P*<0.01
GLY	–22.7%	*P*<0.05
DZX	+12.3%	*P*<0.0001

Control represents no modifiers.

### The effect of changing ER membrane osmolarity on net Ca^2+^ uptake in rat liver microsomes

Osmolality can influence membrane fluidity [10]. The effect of DHA or ARA (both at 50 μM) under different assay osmolarities was examined. To mimic hyperosmolar conditions, the assay contained 100 mM KCl and 154 mM NaCl. And for hypo-osmolarity the KCl concentration was lowered to 50 mM, and no NaCl was present. Control assay conditions were 100 mM KCl and NaCl was absent. Thus, there is a five-fold difference between the hypo- and hyper-osmolarity assay conditions. Net Ca^2+^ uptake in the microsomes was not significantly affected by hypo- or hyper-osmolarity conditions [30].

In addition, after modifying the membrane cholesterol concentration, the effect of DHA or ARA at 50 μM on calcium reuptake was re-examined. MBC was added at 25 mM to decrease cholesterol concentration, while 1.5mM cholesterol with the MBC was added to increase this membrane lipid component [31]. Net Ca^2+^ uptake was not significantly affected by an increase or decrease following these adjustments in membrane cholesterol concentration.

**Figure 5:**
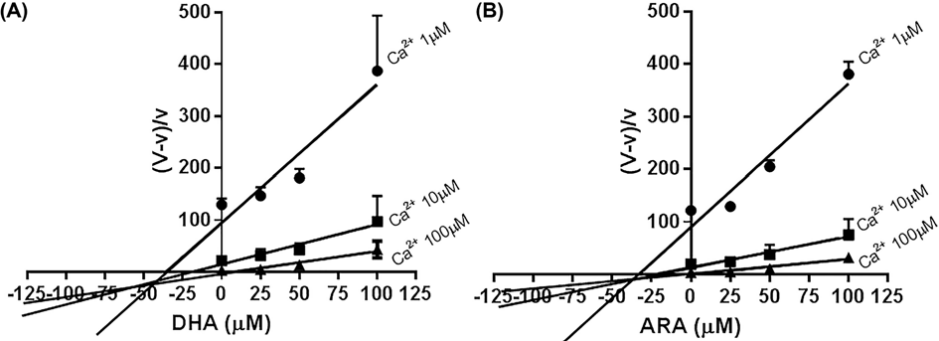
Quotient velocity plots of Ca^2+^ influx velocity in the presence of DHA (**A**) and ARA (**B**) This graphic method can determine both the inhibition constant (K_i_) and the type of inhibition [32]; V = maximal velocity, v = initial velocity, and K_i_ represents the inhibitor constant. Isolated hepatic microsomes were incubated with the indicated FFA and then influx activity measured as described in ‘Materials and methods’ section. The concentration of the FFA ranged from 0 to 100 μM, whereas free calcium was from 1 to 100 μM. The quotient velocity plots were generated based on the fact that ER Ca^2+^ uptake occurs primarily through an embedded membrane enzyme, calcium ATPase. Using this type of plot, the intersection of the lines represents the K_i_ (approximately 40 μM). Of note, this value is very close to the calcium uptake inhibition (IC_50_) by DHA and ARA as depicted in Figure 2.

Quotient velocity plots [32] were created for Ca^2+^ uptake with DHA (Figure 5A) and ARA (Figure 5B). These figures indicate that these PUFAs are mixed type inhibitors of net ER Ca^2+^ accretion (Figure 5A,B). It is reassuring that the K_i_ determined by the intersection was consistent with what is depicted in Figure 2.

### The effect of PUFA electrochemical charge on net Ca^2+^ uptake in rat liver microsomes

**Figure 6:**
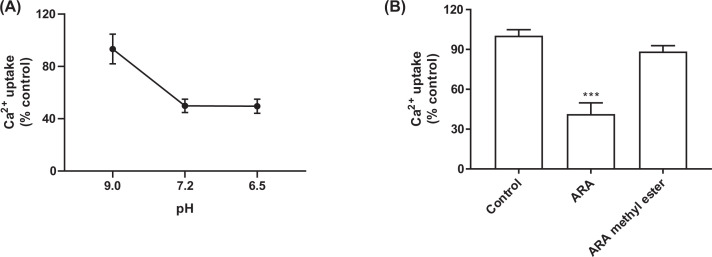
The effect of pH on inhibition by 50 mcM DHA on net calcium uptake in rat hepatic microsomes (A) The effect of ARA, and of an uncharged ARA analog, on net calcium uptake (B) In both figures, 100 μM free calcium and 0.1 μCi/ml of ^45^CaCl_2_ in were used a total volume of 0.4 ml at 37°C and initiated with the addition of the microsomes to a concentration of 0.5 mg/ml and reaction time was 30 min. Data are presented as mean ± SEM (*n*=3). ***P<0.0005 vs control. Control (represented as a 100% in the above figures) was 4.86 ± 0.26 (SEM) nmol/min/mg protein. (**A**) The effect of pH on inhibition by 50 μM DHA on net calcium uptake in rat hepatic microsomes: The effect of DHA 50 μM on Ca^2+^ uptake inhibition under acidic, neutral, and basic pH values using buffer pH of 6.5, 7.2, and 9.0, respectively. Figure shows diminishing inhibition with higher pH values. (**B**) The effect of ARA, and of an uncharged ARA analog, on net calcium uptake: The uncharged methyl ester of ARA failed to inhibit Ca^2+^ uptake.

Unsaturated long-chain fatty acids containing negatively charged phosphate moieties can engage with voltage-gated ion channels [33]. The FFA effect of Ca^2+^ uptake inhibition was studied under different assay conditions which may alter the net negativity of PUFA. Under neutral pH, PUFA is negatively charged, and the net charge depends on the pH [34]. As the pH increases, the net charge in PUFA becomes more negative [35]. We examined the effect of DHA 50 μM on Ca^2+^ uptake inhibition under acidic, neutral, and basic pH values using buffer pH of 6.5, 7.2, and 9.0, respectively. Figure 6 depicts diminishing inhibition with higher pH values. The uncharged methyl ester of ARA failed to inhibit Ca^2+^ uptake. Although not shown, the baseline Ca^2+^ uptake was marginally pH dependent—pH only affected the degree of inhibition by the FFA.

## Discussion

The two essential fatty acids, DHA and ARA, ω-6 and ω-3, respectively, are abundant PUFA in many cellular and organelle membranes.

The PUFA concentrations in our studies are within the range measured in rat plasma. Although unsaturated plasma long-chain FFA in rats on a standard diet can be up to 600 μM (for example, C20:4, n-6), or even higher, in some studies [36,37], there is no information on their intracellular (cytosolic) levels. In humans, the mean plasma n-3 PUFA concentrations are greater than 350 μM, while mean n-6 PUFA concentrations are ten-folds higher [38,39]. Yet some human studies have reported plasma concentration of DHA and ARA at 80.5 and 65.5 μM, respectively [40]. Notwithstanding that the measured total (bound and unbound) PUFA concentrations in plasma are in the μM range, free concentration in tissues are likely lower due to multiple factors such as pH, protein binding, and the ionic milieu [41]. Embedded in the membrane or free in the extracellular space, PUFA may modify the topology of membrane-bound receptors and enzymes and this action may have unsalutary effects [41].

Our data collaborate earlier studies that unsaturated FFA attenuate ER calcium ingress far greater than the saturated fatty acids [20,42]. However, these studies were conducted with sarcoplasmic reticulum vesicles, not hepatic microsomes [20,42]. Also, in agreement with other reports, the inhibitory effect of FFA on ER calcium fluxes or voltage-gated cation channels did not affect endoplasmic ATPase activity [20,43]. Analogously, in cell membranes from rabbit kidney cells, only unsaturated FFA can inhibit both voltage-gated ion channels and Na^+^, K^+^-ATPase activity [44,45].

FFA can beget ER stress and apoptosis in hepatocytes. Disruption of ER homeostasis activates a highly coordinated, adaptive unfolded protein response (UPR), resulting in up-regulation of protein folding capacity and degradation pathways. If this corrective response is overwhelmed, normal protein synthesis is disrupted, and apoptosis may ensue.

To date, the mechanisms that drive ER stress and activation of the UPR to FFA in hepatocytes are speculative. Depletion of ER luminal calcium stores by saturated fatty acids can induce ER stress [3,46]. Given that our study showed no effect of unsaturated FFA on ER Ca^2+^ efflux, yet net Ca^2+^ ER uptake was attenuated, infers that the latter process, possibly through SERCA was affected. However, we found no significant inhibition of hepatic microsomal Ca^2+^-ATPase under our assay conditions, an unexplained peculiarity reported in other studies on FFA inhibition of endoplasmic ion fluxes [19,47]. Earlier studies confirmed the leaky nature of the ER membrane to small molecules (400–500 Da), likely due to a unique lipid bilayer compared with other cell and organelle membranes. This ER membrane leakage may be secondary to a high protein and low cholesterol content, the latter known to fill gaps in the lipid bilayer [48].

Similar to our study, in cell membranes, alterations in fluidity by adjusting cholesterol content did not modify PUFA inhibition of voltage-gated channels [49–51]. In cardiac myocytes, ω-3 fatty acids attenuate calcium influx across the plasma membrane by directly interacting with the calcium channel [25]. Similarly, by directly binding to purified rabbit kidney Na^+^, K^+^-ATPase, unsaturated FFA directly inhibited enzyme activity, whereas saturated FFA had no effect, as was the case in our experiments [45]. Furthermore, the FFA concentration range was 10–100 μM in these experiments, corresponding to concentrations in our hepatic microsome studies. Arguments supporting a direct binding effect of FFA to a channel protein, or an accessory protein, are discussed in detail elsewhere [34,51,52].

The endoplasmic membrane also harbors other ion channels beside calcium. Although well-described in sarcoplasmic reticulum, voltage-gated and inwardly rectifying K_ATP_ channels are likewise found in liver ER. Analogous to cell membrane voltage-gated K^+^ channels, it is plausible that unsaturated FFA in our experiment engage with neighboring ER ion channels, besides Ca^2+^ ATPase, which impact calcium uptake. We observed that inhibition of Ca^2+^ uptake following blockage of the potassium channel was slightly additive to that seen with FFA alone—suggesting a different sites of action of FFA and glibenenclamide. This may occur via ‘lipoelectric modification’ in which a K^+^-voltage sensor is adjusted by electrostatic interaction negatively charged FFA bound to the membrane [33,53]. And by acting through counter current electrochemical forces, modification of these K^+^ channels in the ER membrane could indirectly bear on ER calcium flux. In sum, given that hepatic microsomal ATPase activity was refractory to FFA inhibition in our studies, it is conceivable that the Ca^2+^ uptake inhibition was secondary to a PUFA action on endoplasmic K^+^ channels that, in turn, negatively impacted cation the Ca^2+^-ATPase channel.

Bear in mind that the p*K*_a_ of long-chain FFA depends on the degree of unsaturation and chain length [35,54]. However, with increasing chain length, the p*K*_a_ drifts upwards. And with increasing double bonds, the p*K*_a_ is lowered which renders the FFA more negatively charged. Of these two opposing influences on p*K*_a_, chain length is not as relevant as the abundance of double bonds. Thus, according to the lipoelectric model, our results are in concordance inasmuch as the degree of unsaturation correlated with the potency of FFA inhibition of ER Ca^2+^ uptake.

In cell membranes, PUFA, or their hydrolysis and oxidation products, can alter the activity of several voltage-gated ion channels [34,51,52]. The mechanism of action is seemingly a direct effect on the channel [34], and not by a non-specific remodeling of membrane fluidity. This tenet is supported by experimental evidence, such as changing the fluidity by varying the cholesterol content does not mitigate the inhibitory PUFA effect. A direct FFA effect on membrane channels is further supported by experiments using single point mutations, low PUFA concentrations, and the rapid onset of action.

In conclusion, unsaturated FFA can inhibit hepatic endoplasmic calcium uptake by means of a mixed-type inhibition, and a negative charge of the carboxyl group along with several double bonds appears to be a sine qua non. And, given that the microsomal Ca^+2^-ATPase activity is impervious to the FFA inhibition, the infers that there may be direct channel binding to the uptake complex separate from the ATP hydrolysis site. Lastly, the ATP-dependent calcium uptake pump may engage with an endoplasmic K_ATP_ channels.

## Data Availability

The data that support the findings of the present study are available from the corresponding author upon reasonable request.

## Abbreviations

ARA: arachidonic acid
DHA: docosahexaenoic acid
ER: endoplasmic reticulum
FFA: free fatty acid
MBC: methyl-b-cyclodextrin
PUFA: polyunsaturated FFA
SERCA: sarcoplasmic reticulum Ca^2+^-ATPase
UPR: unfolded protein response
